# New mitochondrial genomes of *Saccostrea* (Mollusca, Ostreidae) specimens from Hainan Island

**DOI:** 10.3897/zookeys.1270.170554

**Published:** 2026-02-18

**Authors:** Dansheng Xie, Fengping Li, Mingjie Liu, Likai Fan, Xingchen Guo, Aimin Wang, Chunsheng Liu, Zhifeng Gu, Yi Yang

**Affiliations:** 1 School of Marine Biology and Fisheries, Hainan University, Haikou 570228, China College of Innovation and Entrepreneurship of Hainan University Haikou China; 2 College of Innovation and Entrepreneurship of Hainan University, Haikou, 570228, China School of Marine Biology and Fisheries, Hainan University Haikou China; 3 Sanya Nanfan Research Institute, Hainan University, Sanya 572025, China Sanya Nanfan Research Institute, Hainan University Sanya China

**Keywords:** Bivalvia, OTUs delimitation, oyster, phylogeny, rock oyster

## Abstract

The taxonomic classification of oysters, particularly within the genus *Saccostrea*, remains poorly resolved due to limitations in traditional morphological and single-gene approaches. To address this, we sequenced the mitochondrial genomes of four *Saccostrea* specimens (*Saccostrea* MBSR 211-214) from Hainan Island. The complete mitochondrial genomes (16,280–16,289 bp) revealed a conserved gene arrangement, including 12 protein-coding genes (PCGs), 23 transfer RNA (tRNA), and two ribosomal RNA (rRNA) genes on a single strand, with a fragmented 16S rRNA gene as a distinctive feature. Different OTU delimitation methods consistently identified these four lineages as two novel operational taxonomic units (OTUs). Within one of the OTUs, there are high levels of intraspecific divergence, exhibiting the potential for independent evolution. Our analysis reclassified the publicly available mitochondrial genomes of *Saccostrea* into nine OTUs, further revealing significant mislabeling on GenBank as indicated by previous studies. The study adds new mitogenomic resources for Saccostrea and provides data useful for future integrative taxonomic work.

## Introduction

The taxonomic classification and phylogenetic relationships of oysters remain unresolved, particularly within the genus *Saccostrea* (Lam and Morton 2006; Sekino and Yamashita 2016; Cui et al. 2021). Traditional morphological approaches have proven problematic, with Stenzel (1971) and Harry (1985) recognizing only a single valid species (*S. cucullata*) within *Saccostrea* for the Indo-Pacific region. However, the phenotypic plasticity of *Saccostrea* varies significantly across different life stages and habitat conditions (Lam and Morton 2006; Reece et al. 2008; McDougall et al. 2024). This plasticity not only leads to substantial intraspecific diversity but also results in extensive interspecific morphological overlap, rendering shell-based taxonomy unreliable (Lam and Morton 2006; McDougall et al. 2024). This complexity is further compounded by frequent interspecific/intraspecific hybridization, introgression events, and the presence of cryptic species complexes, all of which pose additional challenges to conventional morphological identification approaches (Sekino and Yamashita 2013; Hamaguchi et al. 2014; Li et al. 2023). DNA sequencing offers a solution to these limitations, providing more robust evidence for oyster classification (Liu et al. 2011).

The taxonomic complexity of *Saccostrea* has been investigated through molecular approaches. Lam and Morton (2006) established a reliable and reproducible framework using 16S rRNA data, identifying two major complexes in the Indo-Pacific: the *S. cucullata* complex (lineages A–G plus *S. kegaki* and *S. glomerata*) and the *S. mordax* complex (lineages A and B). Subsequent studies by Sekino and Yamashita (2013) recognized *S. palmula* and three *S. mordax* lineages (A–C) from Okinawa Island. Hamaguchi et al. (2014) revised the taxonomic assignment of *S. glomerata* to Japanese specimens, asserting that most populations should be classified as *S. cucullata*, except for *S. kegaki*, *S. mordax*, and *S. mytiloides* and two lineages tentatively assigned to *S. malabonensis*. The Caribbean populations reveal further complexity (Lohan et al. 2015), while Sekino and Yamashita (2016) identified seven mitochondrial lineages (C, F, G, H, I, J, plus *S. kegaki*), although only three showed clear reproductive isolation. Li et al. (2017) resolved taxonomic ambiguities by demonstrating conspecificity between Myanmar populations and distinct reference species, advocating for the recognition of *S. malabonensis* as a valid species. Cui et al. (2021) contributed to this framework by formally describing a new species (*S. mordoides*) from the *S. mordax* complex in southern China. Collectively, these studies highlight the limitations of single-gene approaches, with Guo et al. (2018) suggesting climatic oscillations drove recent speciation events in these oyster complexes.

The tropical western Pacific Ocean represents a global biodiversity hotspot for oysters, distinguished by both the youngest fossil records and the highest species richness among extant oyster populations, especially for *Saccostrea* (Li et al. 2021). Within this biogeographically significant region, Hainan Island’s diverse coastal environments, exceptional water quality, and abundant oyster resources (Lu et al. 2024) provide an ideal natural laboratory for evolutionary studies. A prior DNA barcoding investigation employing COI sequences by Heng et al. (2025) revealed multiple phylogenetically distinct lineages within *Saccostrea*. These lineages exhibited consistent genetic divergence (typically > 2%) when compared to existing *Saccostrea* COI sequences in their investigation or deposited in GenBank, suggesting substantial underestimation of the *Saccostrea* diversity. However, the definitive taxonomic classification of these lineages remained unresolved due to the analytical constraints inherent to single-gene barcoding approaches. Given the inherent limitations of single-gene markers in resolving these issues, advanced molecular genetic tools have become essential for precise species delineation. Oyster mitochondrial genomes, characterized by their rapid evolutionary rates, conserved structural architecture, and strict maternal inheritance pattern, have emerged as particularly valuable molecular markers for systematic studies (Ren et al. 2010; Wu et al. 2010; Yu and Li 2012). The ongoing decline in high-throughput sequencing costs has facilitated a marked increase in the availability of molluscan mitochondrial genome data in recent years. To address this knowledge gap, our study sequenced complete mitochondrial genomes from four *Saccostrea* specimens collected from Hainan Island. Through integrated analyses of mitochondrial genomic architecture and genus-level phylogenetic reconstruction, we re-evaluate the diversity of *Saccostrea* and establish the taxonomic validity of these evolutionarily significant specimens.

## Materials and methods

### Sample collection and DNA extraction

Four specimens representing four *Saccostrea* individuals (designated as *Saccostrea* MBSR 211–214) were selected based on the DNA barcoding study by Heng et al. (2025). The specimens were collected from different locations in Hainan Province, China: *Saccostrea* MBSR-211 from Chengmai (19°59'15"N, 109°53'3"E), *Saccostrea* MBSR-212 from Danzhou (19°31'15"N, 108°56'58"E), *Saccostrea* MBSR-213 from Changjiang (19°28'19"N, 108°53'33"E), and *Saccostrea* MBSR-214 from Lingshui (18°24'19"N, 110°4'7"E). After morphological identification, adductor-muscle tissues were removed and preserved in 95% ethanol. Approximately 25–30 mg of muscle tissue was used for genomic DNA extraction with the TIANGEN Marine Animal DNA Kit (TIANGEN, China). DNA quality was evaluated via 1.0% agarose gel electrophoresis, and qualified samples were subsequently sent to Novogene in Beijing for high-throughput sequencing.

### Illumina sequencing and sequence assembly

DNA libraries with insert fragments of about 300 bp were created using the NEB Next® Ultra™ DNA Library Prep Kit (NEB, USA) following the manufacturer’s instructions. Sequencing was performed on the Illumina NovaSeq 6000 platform, yielding 150 base pair paired-end reads. Quality control of the raw data was conducted using Fastp (Chen et al. 2018), with the adapters and low-quality reads removed. Subsequently, the clean data were imported into Geneious Prime 2025.0.2 (Kearse et al. 2012) to assemble the mitochondrial genomes, with “*S. cuccullata*” (KP967577) as the reference template.

### Mitochondrial genome annotation and basic feature analysis

The mitochondrial protein-coding genes (PCGs) were predicted using MITOS WebServer (Bernt et al. 2013) and ORF Finder (Rombel et al. 2002) with the invertebrate genetic code table. The ribosomal RNA (rRNA) and transfer RNA (tRNA) genes were identified using WebServer​and (Laslett and Canbäck 2008), respectively. The secondary structures of predicted tRNAs were visualized using ​(Darty et al. 2009). The nucleotide composition of the mitochondrial genome, including PCGs, tRNAs, and rRNA genes, was calculated using MEGA 11 (Tamura et al. 2021). Base skew values for a given strand were calculated as follows: AT skew = (A − T) / (A + T), GC skew = (G − C) / (G + C), where A, T, G, and C represented the counts of the four nucleotides. Codon usage in PCGs was analyzed using MEGA 11 with the invertebrate genetic code table. Additionally, the values of Ka, Ka and Ka/Ks ratios among the four species were calculated using DnaSP 6 (Rozas et al. 2017).

### Phylogeny and OTU delimitation of the genus *Saccostrea*

To determine the systematic status of the four specimens, all *Saccostrea* mitochondrial genomes available on GenBank were included for phylogenetic analyses. Based on previous results (e.g. Cui et al. 2021), *S. mordax* was set as the root of the phylogenetic tree. The nucleotide sequences of 12 PCGs were codon-aligned and concatenated for subsequent analyses. The optimal partitioning scheme and corresponding evolutionary models were determined using PartitionFinder 2 (Lanfear et al. 2016). Bayesian inference (BI) was implemented using MrBayes 3.2.6 (Ronquist et al. 2012), running Markov chain Monte Carlo (MCMC) simulations for 10 million generations with sampling every 1,000 generations. The first 25% of samples were discarded as burn-in. Maximum-likelihood (ML) analysis was conducted using IQ - TREE 1.6.12 (Nguyen et al. 2014), allowing partitions to have different evolutionary rates and performing 10,000 ultrafast bootstrap replicates. The resulting phylogenetic trees were visualized using FigTree 1.4.5. The nucleotide sequences of 12 PCGs of *Saccostrea* from phylogenetic analysis was uploaded to the online ASAP (https://bioinfo.mnhn.fr/abi/public/asap/asapold.html) platform for OTU delimitation, with the K80 model selected and other parameters set to default (Puillandre et al. 2021). The ML tree was uploaded to the mPTP online server (https://mcmc-mptp.h-its.org/) to perform OTU delineation under default parameters (Kapli et al. 2017). Subsequently, genetic distances between different OTU groups were calculated using the K80 model in MEGA 11.

## Results

### Mitochondrial genome characteristics

**Figure 1.**
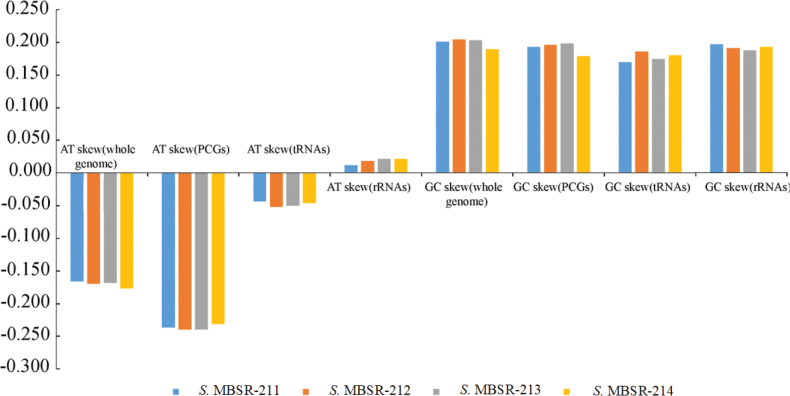
Variations in base composition of the mitochondrial genomes of four *Saccostrea* specimens.

**Figure 2.**
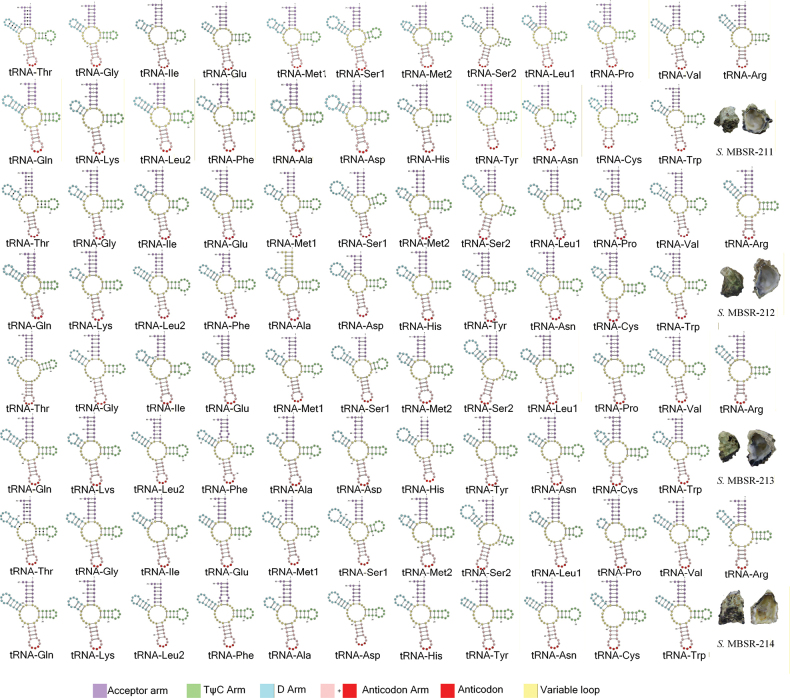
Inferred secondary structures of 23 tRNA genes in four mitochondrial genomes.

**Figure 3.**
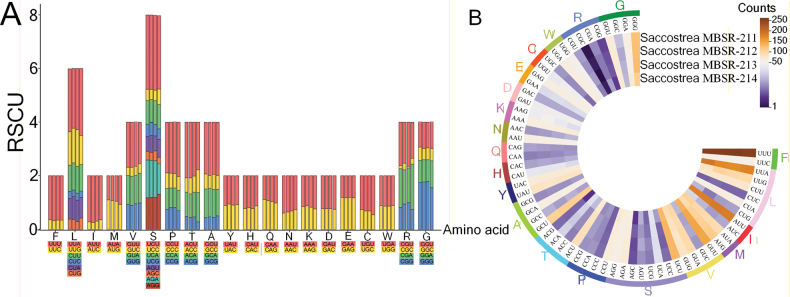
**A**. Relative Synonymous Codon Usage (RSCU); **B**. Frequency of four mitochondrial genomes.

**Figure 4.**
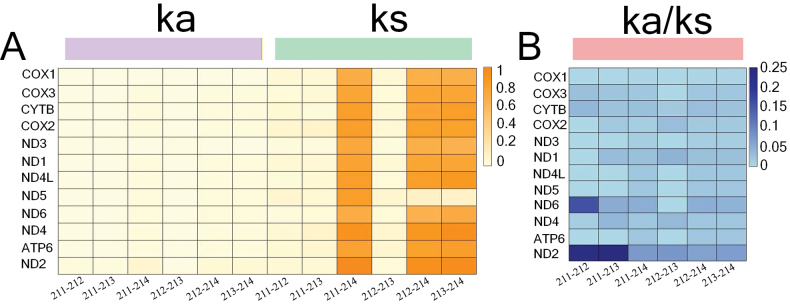
**A**. Values of non-synonymous (Ka) and synonymous (Ks) substitution; **B**. Ka/Ks ratios of each PCG.

The complete mitochondrial genomes of four *Saccostrea* specimens were successfully assembled, with sizes ranging from 16,280 bp (*Saccostrea* MBSR-214) to 16,289 bp (*Saccostrea* MBSR-212). All four mitogenomes exhibit identical gene organization, encoding 12 PCGs, 23 tRNAs, and 2 rRNAs on a single strand, with the distinctive feature of a fragmented 16S rRNA gene (Suppl. material 1: tables S1–S4). Comparative analyses revealed consistent nucleotide composition biases across genomic elements (Fig. 1). AT-skew values demonstrated thymine preference in whole genomes (−0.044 to −0.240), PCGs, and tRNAs, while rRNA genes showed adenine bias (0.011–0.021). All genomic components exhibited guanine preference in GC-skew (0.196–0.204). Secondary structure prediction confirmed that all tRNAs maintain canonical cloverleaf conformations, including the duplicated tRNA-Met, tRNA-Ser, and tRNA-Leu genes (Fig. 2). Initiation codon analysis showed that most PCGs employed ATG start codons, with exceptions including GTG-initiated ND3, ND5, and ND2 (except *Saccostrea* MBSR-214 ND2 which used ATG), and ATA-initiated CYTB. All PCGs terminated with complete stop codons (TAA in 18 genes; TAG in 30 genes). The four species displayed nearly identical codon usage patterns (Fig. 3), with UUU, UUA, UUG, AUU, AUG, GUU, UCU, GCU, and GGG being predominant codons and CGC being the rarest. Leucine represented the most abundant amino acid, while glutamate was the least frequent. Evolutionary analysis indicated limited nonsynonymous substitutions among the four species, with most variations being synonymous. The COX1 gene exhibited the strongest purifying selection (average Ka/Ks = 0.0026), while ND2 and ND6 showed relatively higher Ka/Ks ratios (Fig. 4).

### Phylogenetic relationships and OTU delimitation

**Figure 5.**
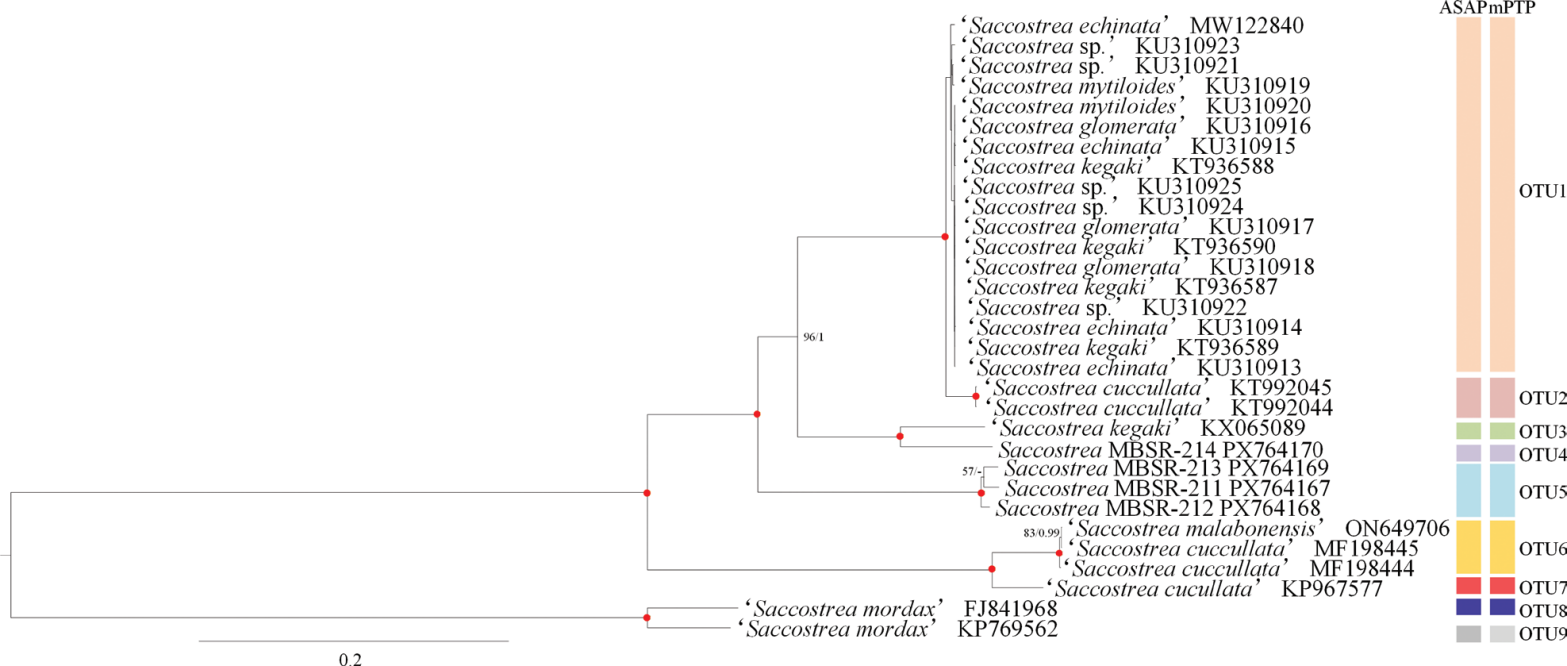
Phylogenetic relationships of *Saccostrea* and species delimitation schemes based on the concatenated nucleotide sequences of 12 mitochondrial protein-coding genes. The first number at each node is bootstrap proportion (BP) of maximum likelihood (ML) analyses, and the second number is Bayesian posterior probability (PP). Nodal with maximum statistical support (BP = 100; PP = 1) is marked with a solid red circle.

Phylogenetic reconstruction based on 12 PCGs from 31 *Saccostrea* mitochondrial genomes yield concordant topologies through ML and BI analyses, consistently resolving five major clades within the genus (Fig. 5). This study employed two delimitation methods (ASAP and mPTP) and identified nine OTUs within the genus *Saccostrea* based on mitochondrial coding sequences. Among these, OTU1, OTU3, OTU6, OTU8, and OTU9 match previously described species (Li et al. 2021; Cui et al. 2021), while the remaining OTUs represent currently unrecognized lineages. Phylogenetically, the nine OTUs are distributed across five major clades. The uppermost clade contains OTU1 and OTU2, which encompass several misannotated *Saccostrea* mitogenomes from GenBank. The second clade consists of sister lineages *S. kegaki* (OTU3) and *Saccostrea* MBSR-214 (OTU4). The third clade includes *Saccostrea* MBSR-211–213, all grouped under OTU5. The fourth clade comprises *S. malabonensis* (OTU6) and OTU7, and the fifth clade corresponds to the *S. mordax* complex (OTU8 and OTU9). Notably, OTU4 and OTU5 show pronounced genetic divergence (>10%) from all other OTUs. Within OTU5, pairwise genetic distances are relatively low: 1.7% between *S.* MBSR-211 and *S.* MBSR-212, 1.6% between *Saccostrea* MBSR-211 and *Saccostrea* MBSR-213, and 1.9% between *Saccostrea* MBSR-212 and *Saccostrea* MBSR-213.

## Discussion

The structural characterization of the four *Saccostrea* mitochondrial genomes obtained in this study aligned with those of other members of the genus. The absence of the ATP8 gene, a common occurrence in bivalves, was also observed here (Zhan et al. 2018; Zhou et al. 2024). Furthermore, the fragmentation of the large subunit 16S rRNA into two segments is a distinctive feature of oysters (Xiao et al. 2015; Milbury et al. 2010). The intergenic region between COX1 and tRNA-Thr exhibited a high AT content, suggesting its putative role as a control region for replication and transcription. Evolutionary analyses revealed strong purifying selection pressures acting on all PCGs, with Ka/Ks ratios consistently below 1.0. This suggests that natural selection favours the preservation of the original gene state, thereby inhibiting the accumulation of mutations (Hurst 2002).

Salvi et al. (2014) first proposed a three-subfamily phylogenetic framework for the family Ostreidae, including Crassostreinae, Saccostreinae, and Ostreinae. Subsequent studies further refined this framework and supported the currently accepted phylogenetic classification of Ostreidae, which includes Crassostreinae (comprising *Crassostrea* Sacco, 1897; *Talonostrea* Li & Qi, 1994; and *Magallana*), Saccostreinae (comprising *Saccostrea* Dollfus & Dautzenberg, 1920), Striostreinae (comprising *Striostrea* Vialov, 1936), and Ostreinae (including the remaining genera) (Raith et al. 2016; Salvi and Mariottini 2016). A characteristic feature of *Saccostrea* is the presence of small chomata along the inner edge of the shell (Lu et al. 2024). Within the genus *Saccostrea*, morphological variation enables further species-level discrimination. The diagnostic criterion for *S. mordax* involves its distinctive wavy shell margins that demonstrate continuous extension into the ligament area, a morphological signature first systematically described by Amaral and Simone (2016). This characteristic feature serves as a reliable morphological marker for distinguishing *S. mordax* from other congeneric species within the genus.

At the molecular level, the two representatives of *S. mordax* are robustly segregated from other *Saccostrea* lineages in the phylogeny, congruent with morphological assessments of *Saccostrea*. Conversely, non-*S. mordax* taxa exhibit pronounced taxonomic incongruence. The pronounced plasticity of oyster shells may mislead non-specialists, resulting in erroneous species assignments and the deposition of mislabelled sequences in GenBank (Cui et al. 2021; Crocetta et al. 2015). For instance, when studying small alien oysters in the Mediterranean, Crocetta et al. (2015) reported that 16S and COI sequences assigned to the genus *Dendostrea* in GenBank did not form a monophyletic clade, with many sequences originating from misidentified specimens. Sequence annotations for the genus *Dendostrea* and related taxa in GenBank are mostly based on shell morphological characteristics and geographic distribution inferences, rather than rigorous integrated molecular and morphological identification, leading to confusion in species delimitation (Oliver et al. 2025; Delcour et al. 2025). Similarly, Sigwart et al. (2021), while describing the new species Crassostrea (Magallana) saidii, confirmed that approximately 5% of COI sequences in the *Magallana* clade were either misidentified or lacked reliable species annotations. Moreover, Hu et al. (2019), in their taxonomic revision of the *Ostrea stentina* species complex, demonstrated that previous sequence annotations of several closely related oysters in GenBank had obscured true species boundaries, underscoring that such classification biases can only be corrected by integrating morphological traits with multilocus molecular data. Historically, oyster systematics relied exclusively on conchological characters. The advent of DNA barcoding subsequently introduced 16S rRNA and COI in contemporary barcoding approaches. Nevertheless, persistent incongruence among morphological, 16S rRNA, and COI datasets creates substantial challenges for achieving unified species delineation. This discordance suggests two opposing systematic issues: potential over splitting leading to synonymy proliferation, versus undetected cryptic diversity masked under single operational taxonomic units (OTUs). Mitogenomes are therefore expected to serve as a powerful tool for data integration across morphological, COI, and 16S datasets.

Notably, within OTU5, pairwise genetic distances range from 1.6% to 1.9%, suggesting that these three samples may represent incipient subspecies or independently evolving specimens (Torstrom et al. 2014; Chen et al. 2024). Indeed, more sensitive delimitation approaches could potentially split OTU5 into three distinct OTUs (data not shown). However, in the absence of robust biological evidence—such as genomic data, geographically distributed populations, or hybridization experiments—we adopt a conservative taxonomic stance and currently treat them as a single OTU. Future studies incorporating broader sampling and multidimensional molecular datasets will be essential to clarify the true evolutionary relationships among these three individuals.

## Conclusion

This study elucidates the complete mitochondrial genomes of four species (*Saccostrea* MBSR-211–214) from Hainan Island, revealing conserved structural features consistent with congeneric organisms​. By integrating phylogenetic reconstruction, and OTU delimitation analysis, it confirms their taxonomic status as distinct specimens within *Saccostrea*. A larger sample set and higher-dimensional molecular data will be essential to clarify the taxonomic status of these lineages. Our findings again underscore the current unreliability of *Saccostrea* nomenclature in public databases such as GenBank. Moving forward, it may be feasible to establish a standardized molecular reference database for oysters through the integrated use of mitochondrial genomes and nuclear gene markers. As biodiversity reassessment progress, enhanced in nomenclature is imperative to avoid taxonomic ambiguities.

## Additional information

### Use of AI

No use of AI was reported.

### Data availability

All of the data that support the findings of this study are available in the main text or Supplementary Information.

## Supplementary materials

Supplementary material 1 Additional information Data type docx Explanation note **table S1**. Gene annotations of the mtDNA of Saccostrea MBSR-211. **table S2**. Gene annotations of the mtDNA of Saccostrea MBSR-212. **table S3**. Gene annotations of the mtDNA of Saccostrea MBSR-213. **table S4**. Gene annotations of the mtDNA of Saccostrea MBSR-214. **table S5**. Genetic distances among different OTUs based on the K80 model. https://binary.pensoft.net/file/1542530This dataset is made available under the Open Database License (http://opendatacommons.org/licenses/odbl/1.0/). The Open Database License (ODbL) is a license agreement intended to allow users to freely share, modify, and use this Dataset while maintaining this same freedom for others, provided that the original source and author(s) are credited. Dansheng Xie, Fengping Li, Mingjie Liu, Likai Fan, Xingchen Guo, Aimin Wang, Chunsheng Liu, Zhifeng Gu, Yi Yang
