# Supplementary material for: New mitochondrial genomes of Saccostrea (Mollusca, Ostreidae) specimens from Hainan Island
Source: Zookeys. 2026 Feb 18;1270:19–31. doi: 10.3897/zookeys.1270.170554 (PMC12936720; doi:10.3897/zookeys.1270.170554)
Supplement: Supplementary material 1 — Additional information [file zookeys-1270-019_article-170554__-s001.docx]

Table S1. Gene annotations of the mtDNA of *Saccostrea* sp.1.

| Gene | From | To | Size(bp) | Start Codon | Stop Codon | Anticodon | Intergenic Nucleotides |
| --- | --- | --- | --- | --- | --- | --- | --- |
| COX1 | 1 | 1656 | 1656 | ATG | TAG |  | 222 |
| tRNA-Thr | 1879 | 1947 | 69 |  |  | UGU | 0 |
| 12S rRNA | 1948 | 2955 | 1008 |  |  |  | 0 |
| tRNA-Gly | 2956 | 3020 | 65 |  |  | UCC | 2 |
| COX3 | 3023 | 3853 | 831 | ATG | TAG |  | 14 |
| tRNA-Ile | 3868 | 3934 | 67 |  |  | GAU | 25 |
| tRNA-Glu | 3960 | 4022 | 63 |  |  | UUC | 89 |
| CYTB | 4112 | 5215 | 1104 | ATA | TAG |  | 11 |
| COX2 | 5227 | 5922 | 696 | ATG | TAG |  | 2 |
| tRNA-Met1 | 5925 | 5994 | 70 |  |  | CAU | 5 |
| tRNA-Ser1 | 6000 | 6070 | 71 |  |  | UGA | 136 |
| tRNA-Met2 | 6207 | 6272 | 66 |  |  | CAU | 7 |
| tRNA-Ser2 | 6280 | 6349 | 70 |  |  | UCU | 1 |
| tRNA-Leu1 | 6351 | 6416 | 66 |  |  | UAA | 4 |
| tRNA-Pro | 6421 | 6483 | 63 |  |  | UGG | 0 |
| 16S rRNA1 | 6484 | 7121 | 638 |  |  |  | 0 |
| tRNA-Val | 7122 | 7189 | 68 |  |  | UAC | 28 |
| tRNA-Arg | 7218 | 7282 | 65 |  |  | UCG | 25 |
| tRNA-Gln | 7308 | 7374 | 67 |  |  | UUG | 3 |
| ND3 | 7378 | 7731 | 354 | GTG | TAA |  | 8 |
| tRNA-Lys | 7740 | 7803 | 64 |  |  | UUU | 18 |
| tRNA-Leu2 | 7822 | 7890 | 69 |  |  | UAG | 0 |
| tRNA-Phe | 7891 | 7958 | 68 |  |  | GAA | 8 |
| tRNA-Ala | 7967 | 8031 | 65 |  |  | UGC | 0 |
| ND1 | 8032 | 8979 | 948 | ATG | TAA |  | 1 |
| ND4L | 8981 | 9262 | 282 | ATG | TAA |  | 12 |
| tRNA-Asp | 9275 | 9341 | 67 |  |  | GUC | 356 |
| 16S rRNA2 | 9698 | 10497 | 800 |  |  |  | 21 |
| ND5 | 10519 | 12189 | 1671 | GTG | TAA |  | 35 |
| ND6 | 12225 | 12689 | 465 | ATG | TAA |  | 11 |
| tRNA-His | 12701 | 12765 | 65 |  |  | GUG | 1 |
| ND4 | 12767 | 14116 | 1350 | ATG | TAA |  | 47 |
| tRNA-Tyr | 14164 | 14227 | 64 |  |  | GUA | 1 |
| ATP6 | 14229 | 14906 | 678 | ATG | TAG |  | 7 |
| tRNA-Asn | 14914 | 14980 | 67 |  |  | GUU | 18 |
| tRNA-Cys | 14999 | 15063 | 65 |  |  | GCA | 5 |
| tRNA-Trp | 15069 | 15137 | 69 |  |  | UCA | 98 |
| ND2 | 15236 | 16240 | 1005 | GTG | TAA |  | 45 |

Table S2. Gene annotations of the mtDNA of *Saccostrea* sp.2.

| Gene | From | To | Size(bp) | Start Codon | Stop Codon | Anticodon | Intergenic Nucleotides |
| --- | --- | --- | --- | --- | --- | --- | --- |
| COX1 | 1 | 1656 | 1656 | ATG | TAG |  | 224 |
| tRNA-Thr | 1881 | 1950 | 70 |  |  | UGU | 0 |
| 12S rRNA | 1951 | 2956 | 1006 |  |  |  | 0 |
| tRNA-Gly | 2957 | 3021 | 65 |  |  | UCC | 2 |
| COX3 | 3024 | 3854 | 831 | ATG | TAG |  | 14 |
| tRNA-Ile | 3869 | 3936 | 68 |  |  | GAU | 25 |
| tRNA-Glu | 3962 | 4024 | 63 |  |  | UUC | 89 |
| CYTB | 4114 | 5217 | 1104 | ATA | TAG |  | 11 |
| COX2 | 5229 | 5924 | 696 | ATG | TAG |  | 2 |
| tRNA-Met | 5927 | 5996 | 70 |  |  | CAU | 5 |
| tRNA-Ser | 6002 | 6072 | 71 |  |  | UGA | 136 |
| tRNA-Met | 6209 | 6273 | 65 |  |  | CAU | 7 |
| tRNA-Ser | 6281 | 6350 | 70 |  |  | UCU | 1 |
| tRNA-Leu | 6352 | 6417 | 66 |  |  | UAA | 4 |
| tRNA-Pro | 6422 | 6484 | 63 |  |  | UGG | 0 |
| 16S rRNA1 | 6485 | 7119 | 635 |  |  |  | 0 |
| tRNA-Val | 7120 | 7190 | 71 |  |  | UAC | 28 |
| tRNA-Arg | 7219 | 7283 | 65 |  |  | UCG | 25 |
| tRNA-Gln | 7309 | 7375 | 67 |  |  | UUG | 3 |
| ND3 | 7379 | 7732 | 354 | GTG | TAA |  | 8 |
| tRNA-Lys | 7741 | 7804 | 64 |  |  | UUU | 18 |
| tRNA-Leu | 7823 | 7891 | 69 |  |  | UAG | 0 |
| tRNA-Phe | 7892 | 7959 | 68 |  |  | GAA | 8 |
| tRNA-Ala | 7968 | 8032 | 65 |  |  | UGC | 0 |
| ND1 | 8033 | 8980 | 948 | ATG | TAA |  | 1 |
| ND4L | 8982 | 9263 | 282 | ATG | TAA |  | 12 |
| tRNA-Asp | 9276 | 9343 | 68 |  |  | GUC | 355 |
| 16S rRNA2 | 9699 | 10498 | 800 |  |  |  | 22 |
| ND5 | 10521 | 12191 | 1671 | GTG | TAG |  | 35 |
| ND6 | 12227 | 12691 | 465 | ATG | TAA |  | 11 |
| tRNA-His | 12703 | 12767 | 65 |  |  | GUG | 1 |
| ND4 | 12769 | 14118 | 1350 | ATG | TAA |  | 49 |
| tRNA-Tyr | 14168 | 14231 | 64 |  |  | GUA | 1 |
| ATP6 | 14233 | 14910 | 678 | ATG | TAG |  | 7 |
| tRNA-Asn | 14918 | 14984 | 67 |  |  | GUU | 18 |
| tRNA-Cys | 15003 | 15067 | 65 |  |  | GCA | 5 |
| tRNA-Trp | 15073 | 15141 | 69 |  |  | UCA | 97 |
| ND2 | 15239 | 16243 | 1005 | GTG | TAG |  | 46 |

Table S3. Gene annotations of the mtDNA of *Saccostrea* sp.3.

| Gene | From | To | Size(bp) | Start Codon | Stop Codon | Anticodon | Intergenic Nucleotides |
| --- | --- | --- | --- | --- | --- | --- | --- |
| COX1 | 1 | 1656 | 1656 | ATG | TAG |  | 221 |
| tRNA-Thr | 1878 | 1946 | 69 |  |  | UGU | 0 |
| 12S rRNA | 1947 | 2953 | 1007 |  |  |  | 0 |
| tRNA-Gly | 2954 | 3018 | 65 |  |  | UCC | 2 |
| COX3 | 3021 | 3851 | 831 | ATG | TAG |  | 14 |
| tRNA-Ile | 3866 | 3934 | 69 |  |  | GAU | 24 |
| tRNA-Glu | 3959 | 4021 | 63 |  |  | UUC | 89 |
| CYTB | 4111 | 5214 | 1104 | ATA | TAG |  | 11 |
| COX2 | 5226 | 5921 | 696 | ATG | TAG |  | 2 |
| tRNA-Met1 | 5924 | 5993 | 70 |  |  | CAU | 5 |
| tRNA-Ser1 | 5999 | 6069 | 71 |  |  | UGA | 136 |
| tRNA-Met1 | 6206 | 6271 | 66 |  |  | CAU | 7 |
| tRNA-Ser2 | 6279 | 6348 | 70 |  |  | UCU | 1 |
| tRNA-Leu1 | 6350 | 6415 | 66 |  |  | UAA | 3 |
| tRNA-Pro | 6419 | 6481 | 63 |  |  | UGG | 0 |
| 16S rRNA1 | 6482 | 7120 | 639 |  |  |  | 0 |
| tRNA-Val | 7121 | 7190 | 70 |  |  | UAC | 27 |
| tRNA-Arg | 7218 | 7282 | 65 |  |  | UCG | 25 |
| tRNA-Gln | 7308 | 7374 | 67 |  |  | UUG | 3 |
| ND3 | 7378 | 7731 | 354 | GTG | TAA |  | 8 |
| tRNA-Lys | 7740 | 7803 | 64 |  |  | UUU | 18 |
| tRNA-Leu2 | 7822 | 7890 | 69 |  |  | UAG | 0 |
| tRNA-Phe | 7891 | 7958 | 68 |  |  | GAA | 8 |
| tRNA-Ala | 7967 | 8031 | 65 |  |  | UGC | 0 |
| ND1 | 8032 | 8979 | 948 | ATG | TAA |  | 1 |
| ND4L | 8981 | 9262 | 282 | ATG | TAG |  | 12 |
| tRNA-Asp | 9275 | 9342 | 68 |  |  | GUC | 355 |
| 16S rRNA2 | 9698 | 10497 | 800 |  |  |  | 21 |
| ND5 | 10519 | 12189 | 1671 | GTG | TAA |  | 35 |
| ND6 | 12225 | 12689 | 465 | ATG | TAA |  | 11 |
| tRNA-His | 12701 | 12765 | 65 |  |  | GUG | 1 |
| ND4 | 12767 | 14116 | 1350 | ATG | TAG |  | 46 |
| tRNA-Tyr | 14163 | 14226 | 64 |  |  | GUA | 1 |
| ATP6 | 14228 | 14905 | 678 | ATG | TAG |  | 7 |
| tRNA-Asn | 14913 | 14979 | 67 |  |  | GUU | 18 |
| tRNA-Cys | 14998 | 15062 | 65 |  |  | GCA | 5 |
| tRNA-Trp | 15068 | 15136 | 69 |  |  | UCA | 97 |
| ND2 | 15234 | 16238 | 1005 | GTG | TAG |  | 46 |

Table S4. Gene annotations of the mtDNA of *Saccostrea* sp.4.

| Gene | From | To | Size(bp) | Start Codon | Stop Codon | Anticodon | Intergenic Nucleotides |
| --- | --- | --- | --- | --- | --- | --- | --- |
| COX1 | 1 | 1656 | 1656 | ATG | TAG |  | 226 |
| tRNA-Thr | 1883 | 1952 | 70 |  |  | UGU | 0 |
| 12S rRNA | 1953 | 2963 | 1011 |  |  |  | 0 |
| tRNA-Gly | 2964 | 3029 | 66 |  |  | UCC | 2 |
| COX3 | 3032 | 3862 | 831 | ATG | TAG |  | 16 |
| tRNA-Ile | 3879 | 3946 | 68 |  |  | GAU | 26 |
| tRNA-Glu | 3973 | 4035 | 63 |  |  | UUC | 91 |
| CYTB | 4127 | 5230 | 1104 | ATA | TAG |  | 15 |
| COX2 | 5246 | 5941 | 696 | ATG | TAG |  | 3 |
| tRNA-Met1 | 5945 | 6014 | 70 |  |  | CAU | 4 |
| tRNA-Ser1 | 6019 | 6089 | 71 |  |  | UGA | 136 |
| tRNA-Met2 | 6226 | 6290 | 65 |  |  | CAU | 9 |
| tRNA-Ser2 | 6300 | 6369 | 70 |  |  | UCU | 1 |
| tRNA-Leu1 | 6371 | 6436 | 66 |  |  | UAA | 4 |
| tRNA-Pro | 6441 | 6502 | 62 |  |  | UGG | 0 |
| 16S rRNA1 | 6503 | 7133 | 631 |  |  |  | 0 |
| tRNA-Val | 7134 | 7201 | 68 |  |  | UAC | 24 |
| tRNA-Arg | 7226 | 7290 | 65 |  |  | UCG | 25 |
| tRNA-Gln | 7316 | 7382 | 67 |  |  | UUG | 3 |
| ND3 | 7386 | 7739 | 354 | ATG | TAG |  | 13 |
| tRNA-Lys | 7753 | 7816 | 64 |  |  | UUU | 17 |
| tRNA-Leu2 | 7834 | 7903 | 70 |  |  | UAG | 0 |
| tRNA-Phe | 7904 | 7971 | 68 |  |  | GAA | 14 |
| tRNA-Ala | 7986 | 8050 | 65 |  |  | UGC | 0 |
| ND1 | 8051 | 8998 | 948 | GTG | TAA |  | 1 |
| ND4L | 9000 | 9281 | 282 | ATG | TAG |  | 13 |
| tRNA-Asp | 9295 | 9362 | 68 |  |  | GUC | 324 |
| 16S rRNA2 | 9687 | 10474 | 788 |  |  |  | 38 |
| ND5 | 10513 | 12183 | 1671 | ATG | TAG |  | 33 |
| ND6 | 12217 | 12681 | 465 | ATG | TAG |  | 21 |
| tRNA-His | 12703 | 12767 | 65 |  |  | GUG | 0 |
| ND4 | 12768 | 14117 | 1350 | ATG | TAA |  | 44 |
| tRNA-Tyr | 14162 | 14224 | 63 |  |  | GUA | 1 |
| ATP6 | 14226 | 14903 | 678 | ATG | TAG |  | 6 |
| tRNA-Asn | 14910 | 14976 | 67 |  |  | GUU | 20 |
| tRNA-Cys | 14997 | 15062 | 66 |  |  | GCA | 6 |
| tRNA-Trp | 15069 | 15134 | 66 |  |  | UCA | 99 |
| ND2 | 15234 | 16238 | 1005 | ATG | TAG |  | 42 |

Table S5. OTU delimitation results of *Saccostrea* by ASAP and mPTP

|  | ASAP | mPTP |
| --- | --- | --- |
| ‘*S. echinata*’ MW122840 | OTU1 | OTU1 |
| ‘*S.* sp.’ KU310923 | OTU1 | OTU1 |
| ‘*S.* sp.’ KU310921 | OTU1 | OTU1 |
| ‘*S. mytiloides*’ KU310919 | OTU1 | OTU1 |
| ‘*S. mytiloides*’ KU310920 | OTU1 | OTU1 |
| ‘*S. glomerata*’ KU310916 | OTU1 | OTU1 |
| ‘*S. echinata*’ KU310915 | OTU1 | OTU1 |
| ‘*S. kegaki*’ KT936588 | OTU1 | OTU1 |
| ‘*S.* sp.’ KU310925 | OTU1 | OTU1 |
| ‘*S.* sp.’ KU310924 | OTU1 | OTU1 |
| ‘*S. glomerata*’ KU310917 | OTU1 | OTU1 |
| ‘*S. kegaki*’ KT936590 | OTU1 | OTU1 |
| ‘*S. glomerata*’ KU310918 | OTU1 | OTU1 |
| ‘*S. kegaki*’ KT936587 | OTU1 | OTU1 |
| ‘*S.* sp.’ KU310922 | OTU1 | OTU1 |
| ‘*S. echinata*’ KU310914 | OTU1 | OTU1 |
| ‘*S. kegaki*’ KT936589 | OTU1 | OTU1 |
| ‘*S. echinata*’ KU310913 | OTU1 | OTU1 |
| ‘*S. cuccullata*’ KT992045 | OTU2 | OTU2 |
| ‘*S. cuccullata*’ KT992044 | OTU2 | OTU2 |
| ‘*S. kegaki*’ KX065089 | OTU3 | OTU3 |
| *S.* sp.4 | OTU4 | OTU4 |
| *S.* sp.3 | OTU5 | OTU5 |
| *S.* sp.1 | OTU5 | OTU5 |
| *S.* sp.2 | OTU5 | OTU5 |
| ‘*S. malabonensis*’ ON649706 | OTU6 | OTU6 |
| ‘*S. cuccullata*’ MF198445 | OTU6 | OTU6 |
| ‘*S. cuccullata*’ MF198444 | OTU6 | OTU6 |
| ‘*S. cucullata*’ KP967577 | OTU7 | OTU7 |
| ‘*S. mordax*’ FJ841968 | OTU8 | OTU8 |
| ‘*S. mordax*’ KP769562 | OTU9 | OTU9 |

Table S6. Genetic distances among different OTUs based on the K80 model

|  | OTU1 | OTU2 | OTU3 | OTU4 | OTU5 | OUT6 | OUT7 | OTU8 | OTU9 |
| --- | --- | --- | --- | --- | --- | --- | --- | --- | --- |
| OTU1 |  |  |  |  |  |  |  |  |  |
| OTU2 | 2.36% |  |  |  |  |  |  |  |  |
| OTU3 | 15.95% | 16.60% |  |  |  |  |  |  |  |
| OTU4 | 15.89% | 16.82% | 10.08% |  |  |  |  |  |  |
| OTU5 | 17.85% | 18.47% | 19.59% | 20.26% |  |  |  |  |  |
| OUT6 | 23.50% | 24.10% | 24.59% | 24.59% | 25.28% |  |  |  |  |
| OUT7 | 22.41% | 23.06% | 24.04% | 24.12% | 24.24% | 7.13% |  |  |  |
| OTU8 | 29.08% | 30.49% | 30.14% | 31.01% | 30.88% | 32.57% | 32.18% |  |  |
| OTU9 | 29.60% | 30.70% | 30.23% | 31.64% | 31.75% | 33.39% | 33.25% | 8.95% |  |
